# ISPC effect is not observed when the word comes too late: a time course analysis

**DOI:** 10.3389/fpsyg.2014.01410

**Published:** 2014-12-05

**Authors:** Nart B. Atalay, Mine Misirlisoy

**Affiliations:** ^1^Department of Psychology, TOBB University of Economics and TechnologyAnkara, Turkey; ^2^Department of Psychology, Middle East Technical UniversityAnkara, Turkey

**Keywords:** ISPC effect, conflict monitoring, contingency learning, stimulus onset asynchrony, Stroop task, cognitive control

## Abstract

The item-specific proportion congruency (ISPC) effect is demonstrated by a smaller Stroop effect observed for mostly incongruent items compared to mostly congruent items. Currently, there is a continuing debate on whether conflict driven item-specific control processes or stimulus-response contingency learning account for the ISPC effect. In the present study, we conducted two experiments to investigate the time course of the ISPC effect with a stimulus onset asynchrony (SOA) manipulation. Both negative and positive SOAs were used in order to manipulate the contingency learning between the word and the color dimensions. We also combined this SOA manipulation with a set size manipulation (Bugg and Hutchison, 2013) to moderate the contribution of contingency learning and item-specific processes to the observed ISPC effect. We expected that the change in the magnitude of the ISPC effect as a result of SOA would follow different patterns for the 2-item and 4-item set conditions. Results showed that the SOA manipulation influenced the ISPC effect. Specifically, when the word followed the color with a 200 ms delay, the observed ISPC effect was smaller, if at all present, than the ISPC effects in other negative and positive SOA conditions, regardless of set size. In conclusion, our results showed that the ISPC effect was not observed if the word arrived too late. We also conducted additional awareness and RT distribution analyses (delta plots) to further investigate the ISPC effect. These analyses showed that a higher percentage of participants were aware of the ISPC manipulation in the 2-item set condition compared to the 4-item set condition. Delta plots revealed that the ISPC effect was smaller for fastest responses and increased as the responses got slower.

## Introduction

Cognitive control is the ability to meet task demands despite distractors and maintain stable performance in the face of changing contexts (Matsumoto and Tanaka, 2004). One of the most commonly used tasks to investigate cognitive control is the Stroop task (Stroop, 1935). In a Stroop task, color words are presented in matching colors (congruent trials) or mismatching colors (incongruent trials) and participants are instructed to name the ink color and ignore the written word. Despite clear instructions, automatic word reading processes interfere with the color naming task, which results in shorter reaction times observed for congruent trials than for incongruent trials. The reaction time difference between the incongruent and the congruent trials is called the Stroop effect. Variations in the magnitude of the Stroop effect have been interpreted as an indication of control over automatic word reading processes. The nature of these control processes have been investigated by observing certain variables and contexts that modulate the Stroop effect.

A widely used example is the list-wide proportion congruency manipulation, in which the magnitude of the Stroop effect is modulated by the proportion of congruent and incongruent trials in a block. Specifically, a larger Stroop effect is observed when the proportion of congruent trials is higher, compared to the condition when the proportion of incongruent trials is higher (Logan and Zbrodoff, 1979; Logan et al., 1984; Tzelgov et al., 1992). These findings were initially attributed to the strategic use of control processes. For instance, the conflict monitoring account presented a mechanistic explanation of how control operations were executed in response to list-wide proportion congruency manipulations (Botvinick et al., 2001; Verguts and Notebaert, 2008). According to this account, an increase in the proportion of incongruent trials in a block resulted in higher levels of conflict, which in turn, increased control over the Stroop effect.

The notion that Stroop effect was controlled by list-wide strategies was challenged by the introduction of the item-specific proportion congruency (ISPC) manipulation by Jacoby et al. (2003). In the ISPC manipulation there were an equal number of congruent and incongruent trials in each block, and the proportion congruency was manipulated at the item level. That is, Jacoby et al. (2003) used two sets of color words (i.e., green and white vs. blue and yellow). The first set of color words were presented mostly in their congruent color (the mostly congruent [MC] condition); the second set of words were presented mostly in their incongruent color (the mostly incongruent [MI] condition). Any observed changes in the magnitude of the Stroop effect in such an experimental design can no longer be attributed to list-wide control processes, since equal number of congruent and incongruent trials in the experiment prevents the participants from predicting the congruency of incoming trials. Their result showed a smaller Stroop effect for the MI items compared to the MC items, which they referred to as the item specific proportion congruency (ISPC) effect.

Jacoby et al. (2003) proposed an item-level reactive control mechanism to explain the ISPC effect. The proposed mechanism consists of a word reading filter which is rapidly triggered by the stimulus feature that predicts proportion congruence. The filter controls the effects of word reading on color naming performance by decreasing the activation for the irrelevant word dimension (Jacoby et al., 1999). This explanation has challenged the classical dichotomy between automatic and controlled processes (Posner and Synder, 1975), by introducing the possibility of *automatic control*. Owing to the demonstration of the ISPC effect, the relative contributions of proactive and reactive control to list level proportion congruency effects have been extensively investigated (Hutchison, 2011; Bugg et al., 2011a; Bugg and Crump, 2012; Abrahamse et al., 2013). It also led to a continuing debate on whether conflict driven cognitive control processes or stimulus-response learning explained the observed ISPC effect.

According to the *conflict monitoring account*, item-specific control is exerted by registering item-specific conflicts (Blais et al., 2007). The conflict monitoring system calculates and keeps records of conflicts for individual items, and modifies the Stroop effect for each item differentially. Conflict-driven control processes are rapidly set following the onset of each stimulus depending on the proportion congruence of items. The assumption that the irrelevant dimension (the word) determines the conflict monitoring and control processes is difficult to accommodate, since it can determine these only after that specific word is read. (Schmidt and Besner, 2008; see also Verguts and Notebaert, 2008; Levin and Tzelgov, 2014).

Schmidt and Besner (2008) challenged the automatic control explanations by showing that proportion congruency in the ISPC design was fully confounded with stimulus-response contingency. They demonstrated that two independent processes, namely, Stroop interference and contingency learning, accounted for the ISPC effect (Schmidt, 2013a,b). One line of support for this claim comes from within- and between language ISPC studies (Atalay and Misirlisoy, 2012; Atalay et al., 2013). Contingency learning effects observed with non-color words under within- and between-language manipulations were parallel to those observed with color words under within- and between-language ISPC manipulations.

Subsequently, Bugg et al., advanced the cognitive control hypothesis by introducing certain boundary conditions for the involvement of control processes in the ISPC effect (Bugg et al., 2011b; Bugg and Hutchison, 2013, Experiments 1-2). They showed that both contingency learning and control processes played a role in the ISPC effect, and that their level of involvement was determined by the degree of efficiency of access to memory representations by the relevant (color) and irrelevant (word) dimensions of the Stroop stimuli. They demonstrated that item-specific control processes were involved when the relevant dimension signaled proportion congruency and/or when the relevant dimension's access to memory representations was strengthened by the experimental manipulation. Otherwise, contingency learning processes came into play; since word-response contingencies were used more readily by participants.

Bugg and Hutchison (2013, Experiment 3) introduced set size as another factor that moderated the contribution of item-specific control to the ISPC effect. They argued that in the classic 2-item set design, a single high-contingency response existed for both MC and MI item sets (see Schmidt and Besner, 2008), which made it possible and advantageous for the participants to rely on a contingency learning mechanism. In order to test this, they introduced a novel 4-item set design. Eight color words were divided into two 4-item sets. In the MC condition, words were presented in their congruent color 80% of the trials. For the remaining 20% of the trials, they were presented in each of the three incongruent colors equally. In the MI condition, words were presented in their congruent color 20% of the trials; for the remaining 80% of the trials, they were presented in each of the three incongruent colors equally. Therefore, a single high-contingency response did not exist for the MI set. In this case, participants were not able to predict the most likely response with high accuracy, in the incongruent trials. This, in turn, promoted the use of item-specific control instead of contingency learning mechanisms.

Bugg and Hutchison (2013) provided two important pieces of evidence supporting their claim. First, they showed that the pattern of the ISPC effects observed for the 2-item and 4-item sets were different. More specifically, in line with the predictions of the contingency account, proportion-congruence effects observed in the congruent and incongruent trials were similar when 2-item sets were used. For the 4-item sets, however, the proportion-congruence effect observed for the incongruent trials were larger than that of the congruent trials. This result would be predicted by the item-specific control account, but not by the contingency account (see Schmidt, 2014 for an alternative view).

The second piece of evidence was obtained by utilizing transfer items, which were introduced in the final block of the experiment. The transfer items were 50% congruent and 50% incongruent. Incongruent transfer items were obtained by choosing MC and MI words equally from the previous (training) blocks and presenting these words with the transfer colors. For the 2-item set condition, RTs for the MC-incongruent and MI-incongruent transfer items were comparable. However, for the 4-item set condition, RTs for the MI-incongruent transfer items were shorter than RTs for the MC-incongruent transfer items. In summary, an ISPC effect was observed with the transfer items in the 4-item set condition, but not in the 2-item set condition. These results showed that item-specific (reactive) control contributed to the ISPC effect even when the word acted as the ISPC signal.

In the present study, our aim was to investigate the time course of the ISPC effect, by using a separated version of the Stroop task, in which stimulus onset asynchrony (SOA) between the word and the color is manipulated (Glaser and Glaser, 1982; Sugg and McDonald, 1994; Appelbaum et al., 2009, 2012; Roelofs, 2010a,b). A well-replicated result was that larger Stroop effects were observed when the color and the word are presented closer in time, compared to when they are more distant. These findings served an important function in testing the models of the Stroop effect (Cohen et al., 1990). In a similar vein, information on the time course of the ISPC effect is expected to help dissociate contingency learning and item-specific control processes underlying the ISPC effect.

**Figure 1:**
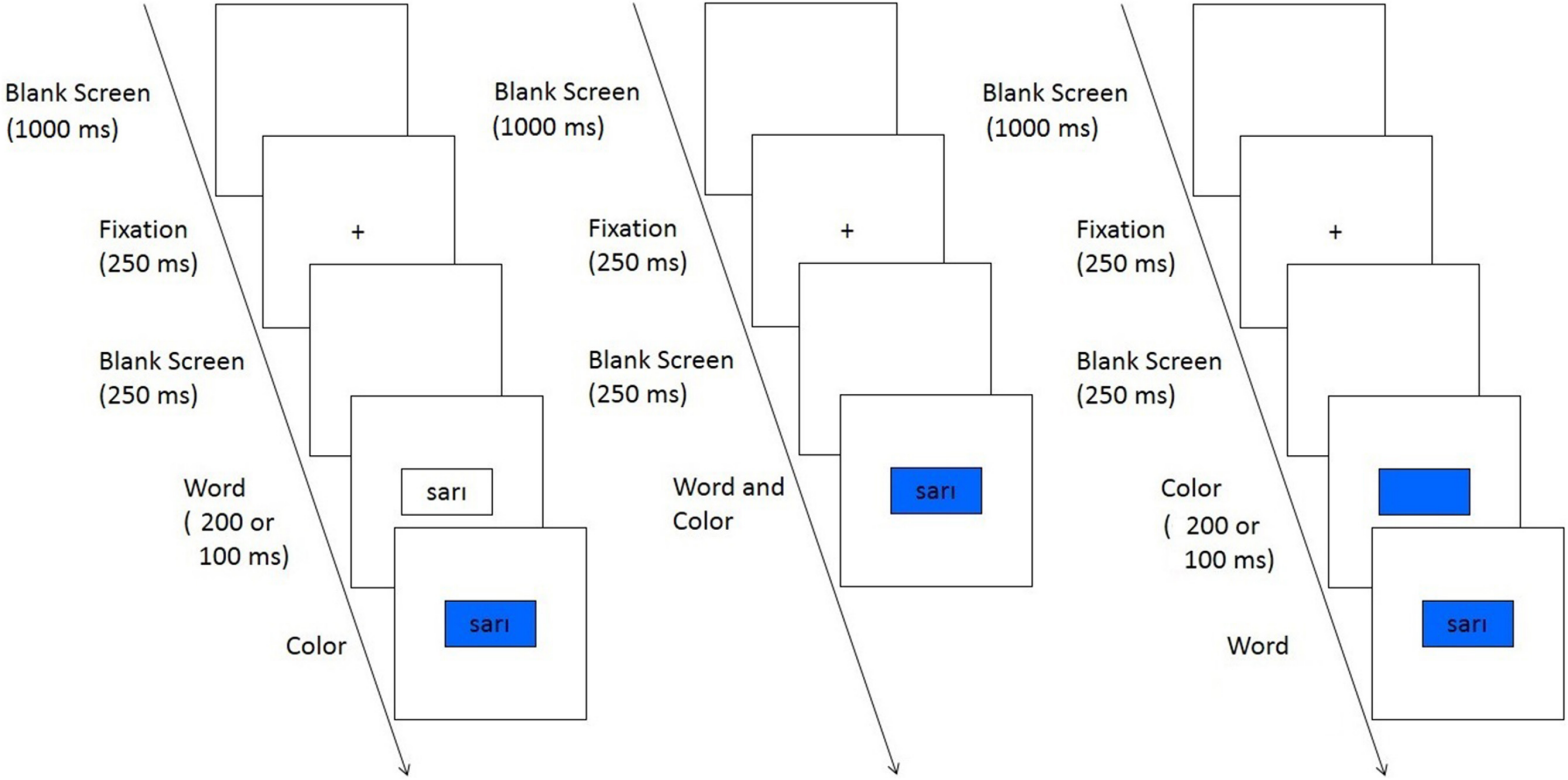
**Trial sequence of Experiments 1 and 2**. Participants named the color of the rectangle. The word appeared before (left), at the same time (middle) or after the colored rectangle (right). The figure is not drawn in scale. Durations are presented in parentheses.

In the present study, we combined the set size manipulation, introduced by Bugg and Hutchison (2013), with an SOA manipulation. The word (the irrelevant dimension) was presented before (−200 ms, −100 ms), simultaneously with (0 ms) or after (+100 ms, +200 ms) the color patch (the relevant dimension, see Figure 1), for both 2-item and 4-item set conditions. We predicted that contingency learning and control processes would be differently affected by the SOA manipulation. In other words, we expected the change in the magnitude of the ISPC effect as a result of the SOA manipulation to follow a different pattern for the 2-item and 4-item set conditions. This resulted in several predictions regarding the level of contribution of control processes and contingency learning to the ISPC effect.

The simultaneous presentation condition, in which the color and the word are presented at the same time, is very similar to the classical color-word Stroop task. Therefore, for the simultaneous presentation condition, in keeping with Bugg and Hutchison (2013), we expected to observe different patterns for the ISPC effects observed in the 2-item and 4-item set conditions. That is, we expected to observe equal proportion congruency effects for the congruent and incongruent trials in the 2-item set condition; which indicate contingency learning processes. In the 4-item set condition, however, the proportion congruency observed for the incongruent trials was expected to be larger than that of the congruent trials; which would indicate control processes.

In the negative SOA conditions, the word was presented before the color. For the 2-item set condition, seeing the word before the color is expected to give participants the opportunity to predict the response even before seeing the color, which would make reliance on contingency learning mechanisms more advantageous. For the negative SOA trials in the 4-item set condition, however, one can predict two different results. On the one hand, the absence of a single high contingency response for incongruent trials in the 4-item set design, together with the presentation of the word before the color, may increase the probability of the word acting as an ISPC signal. That is, item specific control operations may be triggered after seeing the word. If this is the case, then the pattern of results observed in the 4-item set design is expected to be different from that observed in the 2-item set design, which indicate control processes. On the other hand, seeing the word before the color could facilitate word reading, and in turn, make it harder to control the effects of word reading on color naming. In this case, the ISPC effect would be smaller, if at all present.

In the positive SOA conditions, the color was presented before the word. In these conditions, while the structure of the 2-item set design allows the participants to rely on a contingency learning mechanism, seeing the color patch before the word reduces the prediction power of the word (cf. Schmidt and De Houwer, 2012, Experiment 3), making a contingency learning strategy less advantageous. Accordingly, in the 2-item set design for positive SOA conditions, the ISPC effect is expected to be smaller, if at all present. For the 4-item set design positive SOA conditions, since the color is presented before the word, participants might initiate the response even before the word has a chance to trigger control processes, which would eliminate the ISPC effect. In this case, the ISPC effect is expected to be smaller, if at all present. On the other hand, presenting the color before the word could modulate the imbalance between color naming and word reading processes regarding access to memory, in favor of color naming. Therefore, the color would act as an ISPC signal. Consequently, control processes would dominate in the 4-item set positive SOA conditions, and an ISPC effect would be observed.

We conducted two ISPC experiments to investigate the above predictions. In the first experiment, set size was manipulated as a between-subjects factor and SOA was manipulated as a within-subject factor. There were five SOA blocks, within which SOA was kept constant. In keeping with our predictions regarding set size and SOA, we expected the negative and positive SOA conditions to favor control or contingency learning processes depending on set size. Considering the possibility that participants might switch between control and contingency learning processes across different SOA blocks in Experiment 1, which could possibly obscure the results, a second experiment in which SOA was manipulated as a between-subjects factor was necessary. Accordingly, in Experiment 2, both set size and SOA were manipulated as between-subjects factors. This also increased the number of stimuli for each SOA condition, improving the validity of observations.

## Materials and methods

### Participants

There were 126 participants in Experiment 1 (mean age = 21.22, 89 females) and 127 participants in Experiment 2 (mean age = 20.96, 105 females). Participants were university students who volunteered for course credit, or monetary compensation (10 TL~$5). For Experiment 2, sample size was determined with the G^*^Power 3 software (Faul et al., 2007) by using the effect size measure (eta squared) reported in Bugg and Hutchison (2013). We used the same sample size for the within subjects experiment, since we were interested in the four-way interaction between set size, SOA, proportion congruency and item type.

Participants who were not native speakers of Turkish, who reported colorblindness, a reading or attentional disability, or who did not follow the experimental protocol were excluded from the analyses. In addition, if the microphone was not triggered for more than 10% of the trials, the data for that participant was excluded. Analyses were conducted with 106 participants for Experiment 1, and with 113 participants for Experiment 2.

### Stimuli and design

The stimuli and procedure were approved by the local human research ethics committee. Stimuli consisted of a color patch (7.1 × 3.3 cm) and a color word in the middle. Eight colors (blue, gray, green, orange, pink, purple, red, yellow) and their corresponding Turkish color words (mavi, gri, yeşil, turuncu, pembe, mor, kırmızı, sarı) were used in both experiments. For each participant, the 2-item or 4-item sets were selected randomly, and each set was randomly assigned to the MC or MI conditions. MC words were presented with a congruent color patch 83% of the trials and with an incongruent color patch for the remaining 17% of the trials. MI words were presented 83% of the trials with an incongruent color patch, and 17% of the trials with a congruent color patch. There were five blocks of 144 trials (720 trials in total). All eight words were presented 18 times in a block (90 times in total). Table 1 presents a sample of the stimuli arrangement in a single block, for both 2-item and 4-item set conditions. In the 2-item set condition, a single color was used to present items in their incongruent form. In the 4-item set condition, however, three different colors were used to present items in their incongruent form.

**Table 1:** **A sample arrangement and frequency of stimuli in a single block in the 2-item and 4-item set conditions**.

		**Word**	**Color**							
			**blue**	**gray**	**green**	**orange**	**pink**	**purple**	**red**	**yellow**
2-item Set	MC	blue	15	3						
		gray	3	15						
		green			15	3				
		orange			3	15				
	MI	pink					3	15		
		purple					15	3		
		red							3	15
		yellow							15	3
4-item Set	MC	blue	15	1	1	1				
		gray	1	15	1	1				
		green	1	1	15	1				
		orange	1	1	1	15				
	MI	pink					3	5	5	5
		purple					5	3	5	5
		red					5	5	3	5
		yellow					5	5	5	3

MC, mostly congruent; MI, mostly incongruent. Assignment of the stimuli to the MC and MI conditions were random for each participant.

A 2 (set size: 2-item vs. 4-item) × 5 (SOA: −200 ms, −100 ms, 0 ms, +100 ms, +200 ms) × 2 (proportion congruency: MC vs. MI) × 2 (item type: congruent vs. incongruent) mixed-design was used in both experiments. In Experiment 1, proportion congruency, item type, and SOA were manipulated within participants; set size was manipulated between participants. SOA was manipulated across blocks. The order of the blocks was counterbalanced across participants with a Latin square design. Transitions between blocks were not obvious to the participants. SOA between the relevant and the irrelevant dimensions were −200, −100, 0, +100 or +200 ms (see Figure 1). The minus sign denotes presentation of the word before the color. Experiment 2 was identical to Experiment 1, except for the SOA manipulation. In Experiment 2, SOA was kept constant across blocks and it was used as a between-subjects factor.

### Procedure

The procedure was the same for Experiment 1 and Experiment 2. Participants completed the experiment individually in a quiet room within approximately 45 min. Half of the participants were assigned to the 2-item set and the other half to the 4-item set ISPC condition. Before the experiment, participants signed the informed consent form, and filled a questionnaire on color blindness, reading and attentional disability and proficiency in Turkish. Automatic stimulus display and data collection were controlled with a PC running E-Prime 2.0 software. Participants were seated at approximately 60 cm from the monitor. They were given verbal and written instructions to name out loud the ink color of the stimulus, as quickly and as accurately as possible, while ignoring the written word.

Trial sequence as a function of SOA is presented in Figure 1. Trials started with a blank screen (1000 ms), followed by a 250-ms fixation display, and another blank screen (250 ms). As depicted in Figure 1, a color patch was presented before (−200 ms and −100 ms conditions), simultaneously with (0 ms condition), or after (+200 ms and +100 ms conditions) a color word. The color was visible for 1500 ms after its onset. A microphone connected to a Serial Response Box detected the voice onset times. The stimulus appeared on the screen until the voice key was tripped, or until the 1500 ms response deadline was reached. Feedback was given when the voice key was not tripped until the response deadline. Responses were recorded with a second microphone. Before the experiment, participants completed 30 training trials.

Immediately after the experiment, participants answered two questions assessing their awareness of the ISPC manipulation. First question (the awareness question) asked whether or not specific words and colors were paired more frequently than others in the experiment. Participants were required to explain their answer if they answered “yes.” They also gave a confidence judgment for their response by either selecting “certain” or “guessed.” For the next question (the matching question), they were given 10 colors and 10 color words presented as two columns and were asked to connect the more frequently paired colors and color words by drawing a line in-between. Eight of the 10 colors and color words were used in the experiment, the remaining two were new. They were encouraged to guess if they were not certain.

After the participants left the laboratory, the experimenter listened to and coded each trial as correct, incorrect or scratch. Trials were coded as scratch if the voice key was not tripped at all or was tripped by noise.

## Results

### Experiment 1

Prior to the analyses, scratch trials and trials with RTs 3 *SD*s above (or below) the mean were removed. Trials sharing any variety of stimulus- and response-features with the previous trial were also removed to exclude effects sequential repetition or alternation of color and/or word dimensions (Mayr et al., 2003; Hommel et al., 2004). Analyses were run with the remaining 74.7% of the trials. Correct RT and proportion of error (PE) data were analyzed with separate 2X5X2X2 mixed-design ANOVAs, in which set size (2-item vs. 4-item set) was a between-subjects factor; SOA (−200, −100, 0, +100 or +200), proportion congruency (MC vs. MI), and item type (congruent vs. incongruent) were within-subject factors. The alpha level was set at 0.05 for all analyses and partial eta squared (η^2^_p_) is reported as the measure of effect size. *F*s are reported with a Greenhouse-Geisser correction. Tables 2, 3 present mean RTs and PEs for the conditions of Experiment 1, respectively. Only the results from the RT analyses are reported, since PEs were low (*M* = 2.3%) and the results of the PE analyses were parallel with that of the RT analyses. Average number (and standard deviation) of correct RTs per cell in Experiment 1 is presented in Table 4.

**Table 2:** **Mean correct RTs (ms) for the conditions of Experiment 1**.

**PC**	**Item type**	**2-item set (*N* = 56)**					**4-item set (*N* = 50)**				
		**−200 ms**	**−100 ms**	**0 ms**	**+100 ms**	**+200 ms**	**−200 ms**	**−100 ms**	**0 ms**	**+100 ms**	**+200 ms**
Mostly congruent	Congruent	594 (9)	606 (10)	646 (9)	664 (10)	680 (10)	549 (10)	557 (11)	584 (9)	612 (10)	614 (11)
	Incongruent	710 (11)	724 (12)	774 (12)	791 (12)	755 (14)	679 (12)	690 (13)	724 (13)	756 (13)	674 (15)
Mostly incongruent	Congruent	621 (11)	641 (12)	684 (11)	691 (11)	701 (13)	565 (12)	589 (13)	609 (11)	629 (11)	609 (14)
	Incongruent	666 (9)	680 (10)	727 (9)	749 (10)	737 (13)	641 (9)	653 (10)	697 (10)	722 (11)	668 (13)
ISPC effect		71	80	85	69	39	54	70	53	52	1
PC effect congruent		27	35	37	27	21	16	33	26	17	−5
PC effect incongruent		44	44	47	42	18	38	37	28	34	6

Standard errors are presented in parenthesis. PC, proportion congruency. ISPC effect = (MC-incongruent—MC-congruent)—(MI-incongruent—MI-congruent). PC effect congruent = MI-congruent—MC-congruent. PC effect incongruent = MC-incongruent—MI-incongruent.

**Table 3:** **Percentage of errors for the conditions of Experiment 1**.

**PC**	**Item Type**	**2-item set (*N* = 56)**					**4-item set (*N* = 50)**				
		**−200 ms**	**−100 ms**	**0 ms**	**+100 ms**	**+200 ms**	**−200 ms**	**−100 ms**	**0 ms**	**+100 ms**	**+200 ms**
Mostly congruent	Congruent	0.3 (0.1)	0.5 (0.2)	0.2 (0.1)	0.3 (0.1)	0.5 (0.1)	0.2 (0.1)	0.5 (0.2)	0.4 (0.1)	0.2 (0.1)	0.2 (0.1)
	Incongruent	4.5 (1.4)	8.1 (1.7)	6 (1.3)	6.8 (1.6)	3.9 (1)	3.7 (1.4)	5.4 (1.7)	4.4 (1.3)	4.3 (1.7)	3.7 (1.1)
Mostly incongruent	Congruent	1.1 (0.3)	0.7 (0.6)	2.1 (0.5)	2.3 (0.6)	1.8 (0.5)	0 (0.3)	1.5 (0.6)	0.7 (0.6)	0.2 (0.6)	0.2 (0.5)
	Incongruent	2.1 (0.6)	2.9 (0.7)	1.7 (0.5)	3.7 (0.7)	2.9 (0.6)	1.8 (0.7)	2.7 (0.7)	3.4 (0.6)	4.2 (0.8)	2.7 (0.6)
ISPC effect		3.2	5.4	6.2	5.1	2.3	1.7	3.7	1.3	0.1	1.0
PC effect congruent		0.8	0.2	1.9	2.0	1.3	−0.2	1.0	0.3	0.0	0.0
PC effect incongruent		2.4	5.2	4.3	3.1	1.0	1.9	2.7	1.0	0.1	1.0

Standard errors are presented in parenthesis. PC, proportion congruency. ISPC effect = (MC-incongruent—MC-congruent)—(MI-incongruent—MI-congruent). PC effect congruent = MI-congruent—MC-congruent. PC effect incongruent = MC-incongruent—MI-incongruent.

**Table 4:** **Average number of correct RTs per cell in Experiment 1, after the exclusion of scratch trials, trials with RTs 3 SDs above (or below) the mean, errors, and trials sharing any variety of stimulus- and response-features with the previous trial**.

**PC**	**Item type**	**2-item set**					**4-item set**				
		**−200 ms**	**−100 ms**	**0 ms**	**+100 ms**	**+200 ms**	**−200 ms**	**−100 ms**	**0 ms**	**+100 ms**	**+200 ms**
Mostly congruent	Congruent	49.8 (3.3)	49.8 (3.4)	49.5 (3.4)	50.1 (2.6)	49.4 (4.7)	50.7 (2.9)	49.8 (3.8)	49.7 (3.3)	49.7 (3.8)	49.9 (2.8)
	Incongruent	8.3 (1.8)	7.7 (1.7)	7.7 (1.7)	7.5 (1.9)	7.6 (1.7)	7.9 (1.8)	7.7 (1.9)	8.1 (1.9)	7.2 (1.9)	7.8 (1.4)
Mostly incongruent	Congruent	8.6 (1.4)	8.8 (1.5)	8.9 (1.6)	8.6 (1.8)	8.3 (1.8)	9.4 (1.3)	8.8 (1.6)	8.9 (1.8)	8.9 (1.7)	9.1 (1.5)
	Incongruent	42.4 (3.9)	41.9 (4.6)	41.9 (4.1)	40.9 (4)	40.6 (4.9)	35.5 (3.6)	34 (3.5)	33.6 (3.9)	31.9 (4.3)	33.3 (3.6)

Standard deviations are presented in parenthesis. Numbers are calculated after the exclusion of scratch trials, trials with RTs 3 SDs above (or below) the mean, errors, and trials sharing any variety of stimulus- and response-features with the previous trial. There were a total of 60 trials in the mostly congruent-congruent and mostly incongruent-incongruent conditions. There were a total of 12 trials in the mostly congruent-incongruent and mostly incongruent-congruent conditions.

**Figure 2:**
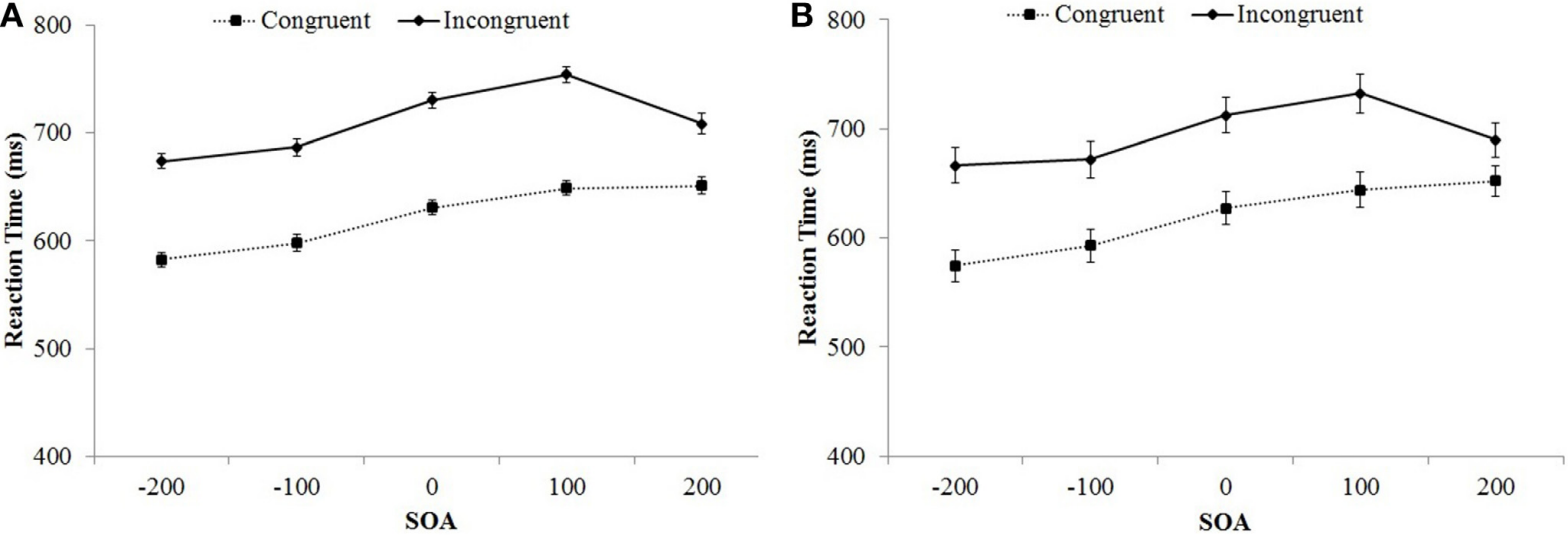
**Mean reaction time as a function of SOA and trial type in Experiment 1 (A) and Experiment 2 (B)**. Bars show standard errors.

**Figure 3:**
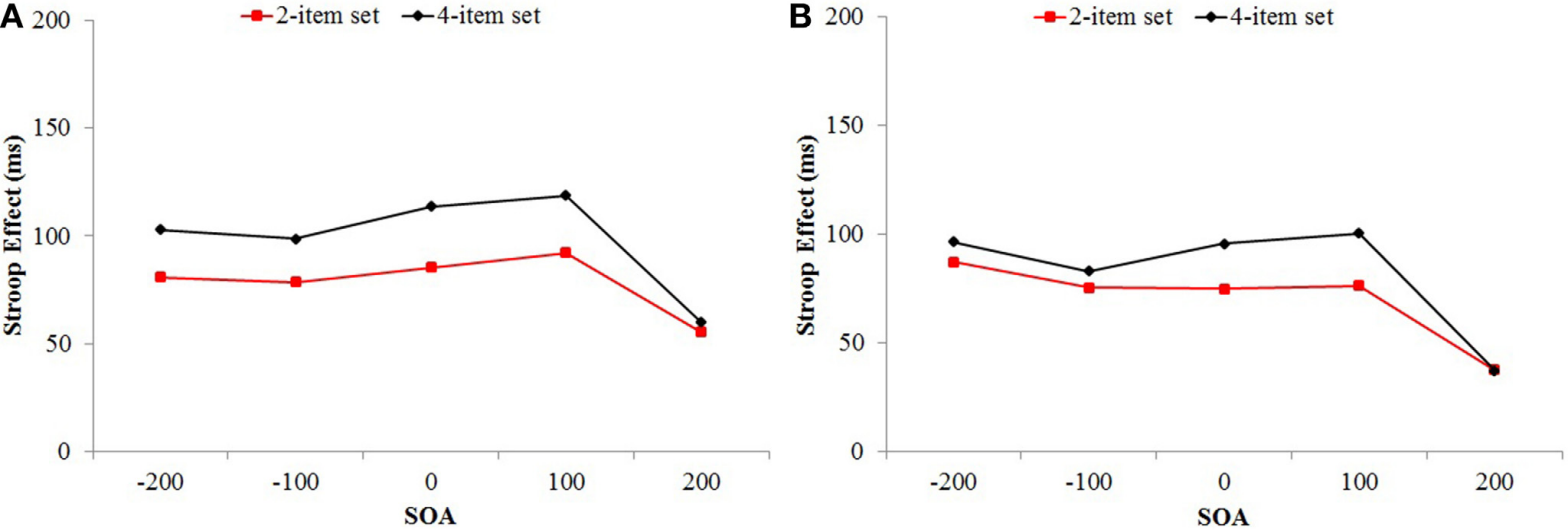
**Mean Stroop effect as a function of set size and SOA in Experiment 1 (A) and Experiment 2 (B)**.

The between-subjects main effect of set size was significant, *F*_(1, 104)_ = 16.56, *MSE* = 82,862, *p* < 0.001, η^2^_p_ = 0.14. Overall, responses were slower for the 2-item sets (692 ms) compared to the 4-item sets (641 ms). There was a significant main effect of SOA, *F*_(3.48, 362.07)_ = 69.14, *MSE* = 6436, *p* < 0.001, η^2^_p_ = 0.40. These main effects were also qualified by a significant set size × SOA interaction, *F*_(3.48, 362.07)_ = 4.61, *MSE* = 6436, *p* = 0.002, η^2^_p_ = 0.04. RTs linearly increased as SOAs become more positive, but there was a decrease in RTs in the +200 condition. This decrease was more pronounced for the 4-item set compared to the 2-item set condition. The Stroop effect (the main effect of item type) was significant, *F*_(1, 104)_ = 733.25, *MSE* = 5653, *p* < 0.001, η^2^_p_ = 0.88, and it significantly interacted with set size, *F*_(1, 104)_ = 9.55, *MSE* = 5653, *p* = 0.003, η^2^_p_ = 0.08. Overall the Stroop effect was smaller for the 2-item set (79 ms) compared to the 4-item set (99 ms) condition. Furthermore, the Stroop effect significantly interacted with SOA *F*_(3.45, 358.77)_ = 22.48, *MSE* = 1882, *p* < 0.001, η^2^_p_ = 0.18. Congruent and incongruent RTs increased steadily as SOAs became more positive, but there was a sharp decrease for the incongruent +200 ms condition (see Figure 2A). The three-way set size × SOA × item-type interaction was not significant, *F* = 1.46. The pattern of change in the Stroop effect across SOA blocks was similar for the 2-item, and the 4-item sets (see Figure 3A).

**Figure 4:**
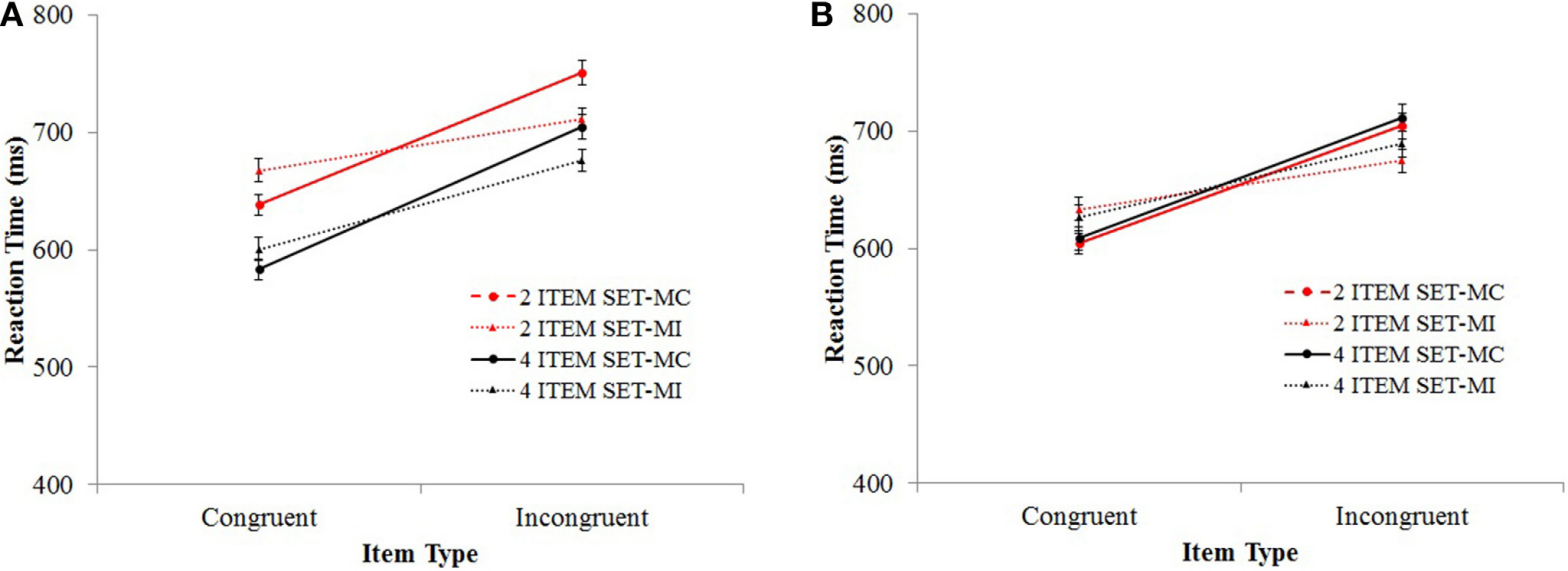
**Mean reaction time a function of set size, proportion congruency and item type in Experiment 1 (A) and Experiment 2 (B)**. Bars show standard errors.

**Figure 5:**
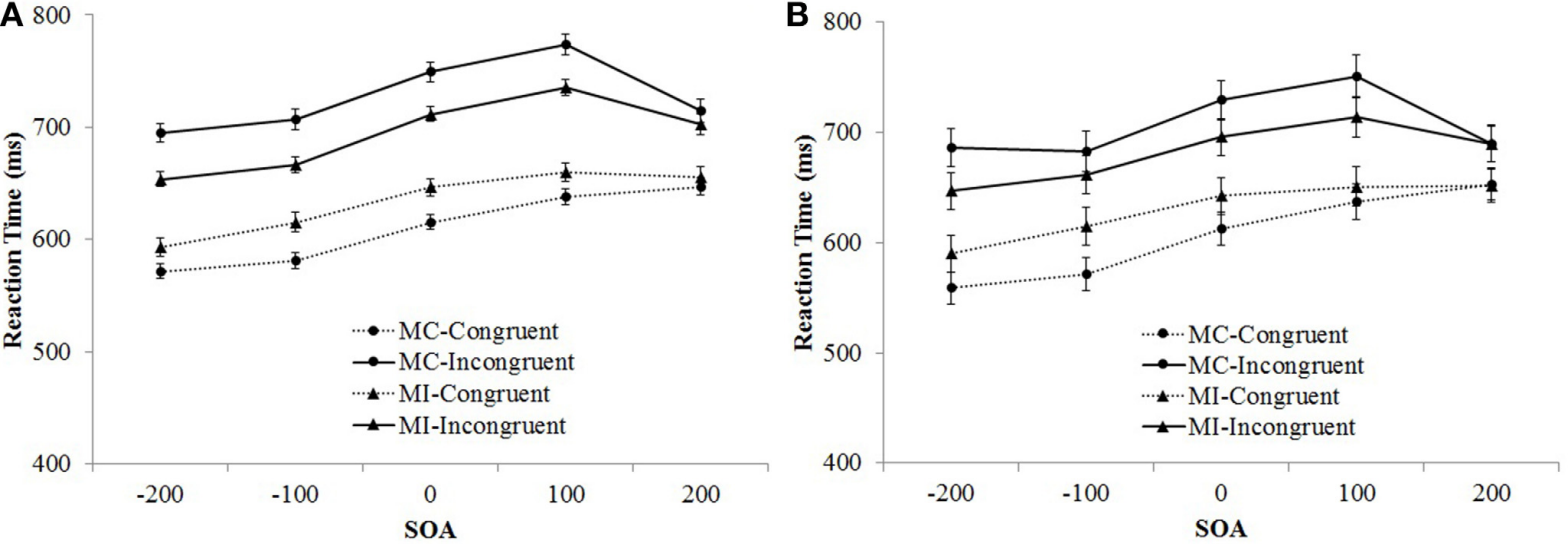
**Mean reaction time a function of SOA, proportion congruency and item type in Experiment 1 (A) and Experiment 2 (B)**. Bars show standard errors.

**Figure 6:**
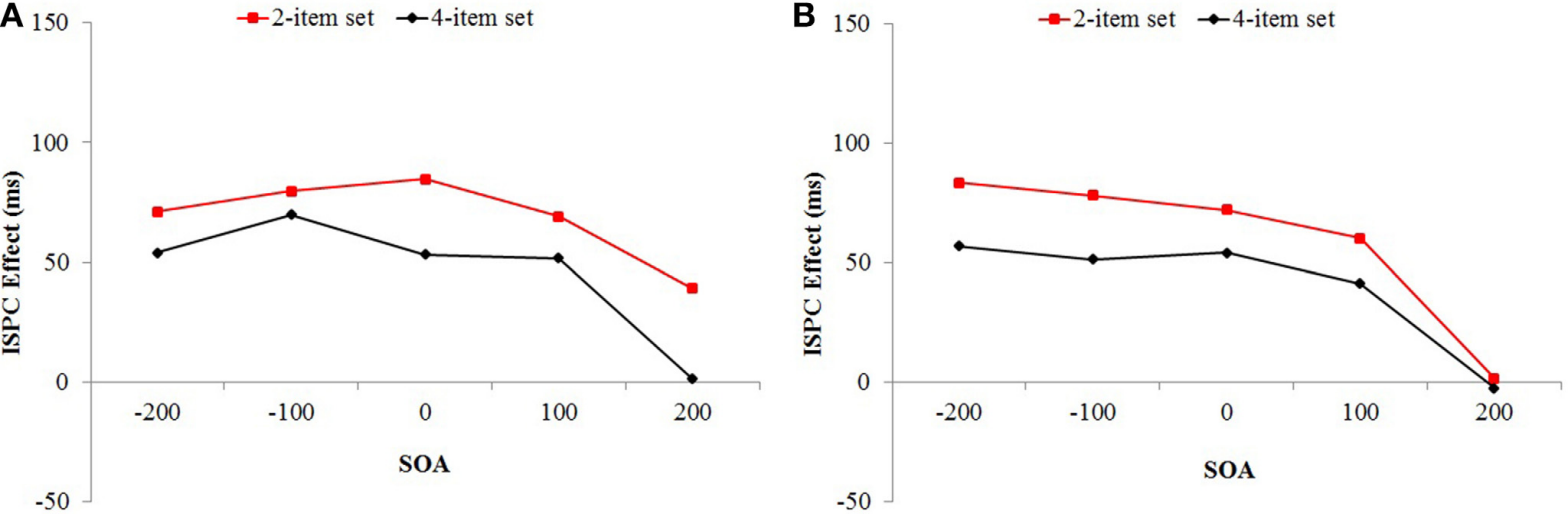
**Mean ISPC effect a function of set size and SOA in Experiment 1 (A) and Experiment 2 (B)**.

The ISPC effect, that is, the two-way interaction between proportion congruency and item type was significant, *F*_(1, 104)_ = 161.07, *MSE* = 2698, *p* < 0.001, η^2^_p_ = 0.61. The three-way interaction between set size, proportion congruency, and item type was also significant, *F*_(1, 104)_ = 6.33, *MSE* = 2698, *p* = 0.013, η^2^_p_ = 0.06. The ISPC effect was larger for the 2-item set (69 ms) compared to the 4-item set condition (46 ms) (see Figure 4A). Important to our study, the ISPC effect changed across SOA conditions, which was indicated by the significant three-way interaction between SOA, proportion congruency, and item type, *F*_(3.79, 394.37)_ = 10.60, *MSE* = 1214, *p* < 0.001, η^2^_p_ = 0.09 (see Figure 5A). The observed ISPC effect was 63, 75, 69, and 60 ms, and 20 ms for the for the −200, −100, 0 +100, and +200 SOA conditions, respectively. Critically, the four-way interaction between set size, SOA, proportion congruency, and item type was not significant, *F* < 1 (see Figure 6A). The relationship between the ISPC effect and SOA was similar for 2-item set and 4-item set conditions.

We ran an additional, 2X4X2X2 mixed-design ANOVA, removing the +200 ms SOA condition, in order to investigate whether interactions involving SOA would remain significant without the +200 ms condition. Therefore, we only report changes involving SOA. First, the two-way interaction between set size and SOA was no longer significant, *F*_(2.68, 278.60)_ = 1.05, *p* = 0.37. Second, the three-way interaction between SOA, proportion congruency, and item type was no longer significant, *F*_(2.89, 300.79)_ = 1.06, *p* = 0.37^1^.

We also compared proportion congruence (PC) effects for incongruent and congruent items across the SOA conditions (including the +200). PC effect for congruent items was calculated as MI-congruent minus MC-congruent. PC effect for incongruent items was calculated as MC-incongruent minus MI-incongruent. PC effects were analyzed with a 2X5X2 mixed-design ANOVA, in which set size (2-item vs. 4-item set) was a between-subjects factor; SOA (−200, −100, 0, +100 or +200), and item type (congruent vs. incongruent) were within-subject factors. There was a main effect of set size, *F*_(1, 104)_ = 6.33, *MSE* = 5396.86, *p* < 0.05, η^2^_p_ = 0.06. There was also a main effect of SOA, *F*_(3.79, 394.37)_ = 10.59, *MSE* = 2428.22, *p* < 0.001, η^2^_p_ = 0.09. Critically, neither the two-way interaction between item-type and set size, *F*_(1, 104)_ = 0.11, *p* = 0.92, nor the two-way interaction between item-type and SOA, *F*_(3.72, 386.62)_ = 1.25, *p* = 0.29, nor the three-way interaction between SOA, item-type and set size, *F*_(3.72, 386.62)_ = 0.45, *p* = 0.76, were significant ^2^.

### Experiment 2

Similar to Experiment 1, scratch trials and trials with RTs 3 *SD*s above (or below) the mean were removed. Trials sharing any variety of stimulus- and response-features with the previous trial were also removed to exclude effects sequential repetition or alternation of color and/or word dimensions (Mayr et al., 2003; Hommel et al., 2004). Analyses were run with the remaining 76% of the trials. Correct RT and PE data were analyzed with separate 2X5X2X2 mixed-design ANOVAs, in which set size (2-item vs. 4-item set) and SOA (−200, −100, 0, +100 or +200) were between-subjects factors; proportion congruency (MC vs. MI), and item type (congruent vs. incongruent) were within-subject factors. The alpha level was set at 0.05 for all analyses and partial eta squared (η^2^_p_) is reported as the measure of effect size. Tables 5, 6 present mean RTs and PEs for the conditions of Experiment 2, respectively. Only the results from the RT analyses are reported, since PEs were low (*M* = 1.9%) and the results of the PE analyses were parallel with that of the RT analyses.

**Table 5:** **Mean correct RTs (ms) for the conditions of Experiment 2**.

**PC**	**Item type**	**2-item set**					**4-item set**				
		**−200 ms (*N* = 12)**	**−100 ms (*N* = 12)**	**0 ms (*N* = 13)**	**+100 ms (*N* = 11)**	**+200 ms (*N* = 13)**	**−200 ms (*N* = 11)**	**−100 ms (*N* = 10)**	**0 ms (*N* = 10)**	**+100 ms (*N* = 9)**	**+200 ms (*N* = 12)**
Mostly congruent	Congruent	563 (20)	557 (20)	600 (19)	655 (21)	646 (19)	555 (21)	585 (22)	625 (22)	619 (23)	659 (20)
	Incongruent	692 (24)	672 (24)	711 (23)	762 (25)	684 (23)	680 (25)	694 (27)	747 (27)	740 (28)	695 (24)
Mostly incongruent	Congruent	598 (23)	606 (23)	628 (22)	693 (24)	643 (22)	582 (24)	624 (25)	656 (25)	608 (27)	661 (23)
	Incongruent	644 (23)	642 (23)	667 (22)	739 (24)	679 (22)	650 (24)	681 (25)	725 (25)	688 (26)	700 (23)
ISPC effect		83	78	72	60	2	57	51	54	41	−3
PC effect congruent		35	49	28	38	−3	27	39	32	−11	2
PC effect incongruent		48	29	44	22	5	30	13	22	52	−5

Standard errors are presented in parenthesis. PC, proportion congruency. ISPC effect = (MC-incongruent—MC-congruent)—(MI-incongruent—MI-congruent). PC effect congruent = MI-congruent—MC-congruent. PC effect incongruent = MC-incongruent—MI-incongruent.

**Table 6:** **Percentage of errors for the conditions of Experiment 2**.

**PC**	**Item type**	**2-item set**					**4-item set**				
		**−200 ms (*N* = 12)**	**−100 ms (*N* = 12)**	**0 ms (*N* = 13)**	**+100 ms (*N* = 11)**	**+200 ms (*N* = 13)**	**−200 ms (*N* = 11)**	**−100 ms (*N* = 10)**	**0 ms (*N* = 10)**	**+100 ms (*N* = 9)**	**+200 ms (*N* = 12)**
Mostly congruent	Congruent	0 (0.1)	0.3 (0.1)	0.1 (0.1)	0.1 (0.1)	0.5 (0.1)	0.2 (0.1)	0.2 (0.2)	0.3 (0.2)	0.1 (0.2)	0.4 (0.1)
	Incongruent	3.6 (1.5)	5.8 (1.5)	5.5 (1.4)	4.5 (1.6)	4.1 (1.4)	1.5 (1.6)	2.9 (1.6)	4.8 (1.6)	6.3 (1.7)	3.2 (1.5)
Mostly incongruent	Congruent	0.2 (0.4)	0.8 (0.4)	0.6 (0.4)	0.2 (0.4)	1.4 (0.4)	0.2 (0.4)	0.7 (0.4)	0.3 (0.4)	0.2 (0.4)	1 (0.4)
	Incongruent	1.3 (0.9)	3.1 (0.9)	2.5 (0.8)	2 (0.9)	3.2 (0.8)	0.5 (0.9)	1.6 (0.9)	4.3 (0.9)	5.4 (1)	2.2 (0.9)
ISPC effect		2.4	3.2	3.5	2.5	1.8	1.0	1.7	0.4	1.0	1.6
PC effect congruent		0.2	0.5	0.5	0.1	0.8	0.0	0.5	−0.1	0.1	0.5
PC effect incongruent		2.3	2.7	3.0	2.5	0.9	1.0	1.2	0.5	0.9	1.1

Standard errors are presented in parenthesis. PC, proportion congruency. ISPC effect = (MC-incongruent—MC-congruent)—(MI-incongruent—MI-congruent). PC effect congruent = MI-congruent—MC-congruent. PC effect incongruent = MC-incongruent—MI-incongruent.

The between-subjects main effect of SOA was significant, *F*_(4, 103)_ = 3.31, *MSE* = 21,641, *p* = 0.014, η^2^_p_ = 0.11. RTs linearly increased as SOAs became more positive. The Stroop effect (the main effect of item type) was significant, *F*_(1, 103)_ = 615.75, *MSE* = 1059, *p* < 0.001, η^2^_p_ = 0.86, and it's interaction with set size was also significant, *F*_(1, 103)_ = 3.95, *MSE* = 1059, *p* = 0.049, η^2^_p_ = 0.04. Overall the Stroop effect was smaller for the 2-item set (70 ms) compared to the 4-item set (83 ms) condition. Furthermore, the interaction between the Stroop effect and SOA was significant *F*_(4, 103)_ = 11.36, *MSE* = 1059, *p* < 0.001, η^2^_p_ = 0.31. Congruent and incongruent RTs increased steadily as SOAs became more positive, but there was a sharp decrease for the incongruent +200 ms condition (see Figure 2B). The three-way set size × SOA × item-type interaction was not significant, *F* < 1. The pattern of change in the Stroop effect across SOA blocks was similar for the 2-item, and the 4-item set conditions (see Figure 3B).

The two-way interaction between proportion congruency and item type (the ISPC effect) was significant, *F*_(1, 103)_ = 162.54, *MSE* = 423, *p* < 0.001, η^2^_p_ = 0.61. ISPC effect and set size interaction was also significant, *F*_(1, 103)_ = 5.86, *MSE* = 423, *p* = 0.017, η^2^_p_ = 0.05. The ISPC effect was larger for the 2-item set (59 ms) compared to the 4-item set (40 ms) condition (see Figure 4B). There was a significant three-way interaction between SOA, proportion congruency, and item type, *F*_(4, 103)_ = 12.04, *MSE* = 423, *p* < 0.001, η^2^_p_ = 0.32 (see Figure 5B). The observed ISPC effect was72, 69, 63, and 56 ms, and −1 ms for the −200, −100, 0 +100, and +200 SOA conditions, respectively. Important for the study, the four-way interaction between set size, SOA, proportion congruency, and item type was not significant, *F* < 1 (see Figure 6B). The relationship between the ISPC effect and SOA was similar for the 2-item set and 4-item set conditions. Pair-wise comparisons of ISPC effects across SOAs showed that only the ISPC effect observed in the +200 ms condition was significantly different from all other SOA conditions. Average number (and standard deviation) of correct RTs per cell in Experiment 2 is presented in Table 7.

**Table 7:** **Average number of correct RTs per cell in Experiment 2**.

**PC**	**Item type**	**2-item set**					**4-item set**				
		**−200 ms**	**−100 ms**	**0 ms**	**+100 ms**	**+200 ms**	**−200 ms**	**−100 ms**	**0 ms**	**+100 ms**	**+200 ms**
Mostly congruent	Congruent	250 (14.5)	251.2 (8.8)	249.8 (6.7)	252.5 (9.8)	248.8 (6.9)	252.6 (7.9)	248.3 (6.3)	253.5 (3.8)	252.1 (7)	251.8 (7.8)
	Incongruent	41.1 (4.4)	41 (5.1)	40.2 (4.2)	39.6 (6.1)	41.8 (4.5)	41.4 (3.5)	41.1 (4.4)	40.3 (6.1)	38.2 (3)	40.2 (7.3)
Mostly incongruent	Congruent	42.3 (3.1)	45.8 (3)	45.8 (4.5)	44 (6)	44.6 (3.7)	45.9 (3)	46.2 (2.8)	45 (4.3)	44.9 (4)	44.6 (3)
	Incongruent	213.5 (8.6)	211 (11.9)	215.6 (11.1)	209.3 (14.3)	213.1 (10.8)	178.9 (10.5)	171.8 (10.4)	165.8 (12.8)	163 (14.3)	172.5 (11.1)

Standard deviations are presented in parenthesis. Numbers are calculated after the exclusion of scratch trials, trials with RTs 3 SDs above (or below) the mean, errors, and trials sharing any variety of stimulus- and response-features with the previous trial. There were a total of 300 trials in the mostly congruent-congruent and mostly incongruent-incongruent conditions. There were a total of 60 trials in the mostly congruent-incongruent and mostly incongruent-congruent conditions.

We ran an additional, 2X4X2X2 mixed-design ANOVA, removing the +200 ms SOA condition, in order to investigate whether interactions involving SOA would remain significant without the +200 ms condition. Therefore, we only report changes involving SOA. First, the two-way interaction between item type and SOA was no longer significant, *F*_(3, 80)_ = 0.57, *p* = 0.64. Second, the three-way interaction between SOA, proportion congruency, and item type was no longer significant, *F*_(3, 80)_ = 0.72, *p* = 0.55.

We also compared proportion congruence (PC) effects for incongruent and congruent items across the SOA conditions (including the +200). PC effect for congruent items was calculated as MI-congruent minus MC-congruent. PC effect for incongruent items was calculated as MC-incongruent minus MI-incongruent. PC effects were analyzed with a 2X5X2 mixed-design ANOVA, in which set size (2-item vs. 4-item set) was a between-subjects factor; SOA (−200, −100, 0, +100 or +200), and item type (congruent vs. incongruent) were within-subject factors. There was a main effect of set size, *F*_(1, 103)_ = 5.86, *MSE* = 846.35, *p* < 0.05, η^2^_p_ = 0.05. There was a main effect of SOA, *F*_(4, 103)_ = 12.04, *MSE* = 846.35, *p* < 0.001, η^2^_p_ = 0.32. Critically, neither the two-way interaction between item-type and set size, *F*_(1, 103)_ = 0.85, *p* = 0.77, nor the two-way interaction between item-type and SOA, *F*_(4, 103)_ = 0.99, *p* = 0.42, nor the three-way interaction between SOA, item-type and set size, *F*_(4, 103)_ = 1.56, *p* = 0.19, were significant.

### Awareness data

**Figure 7:**
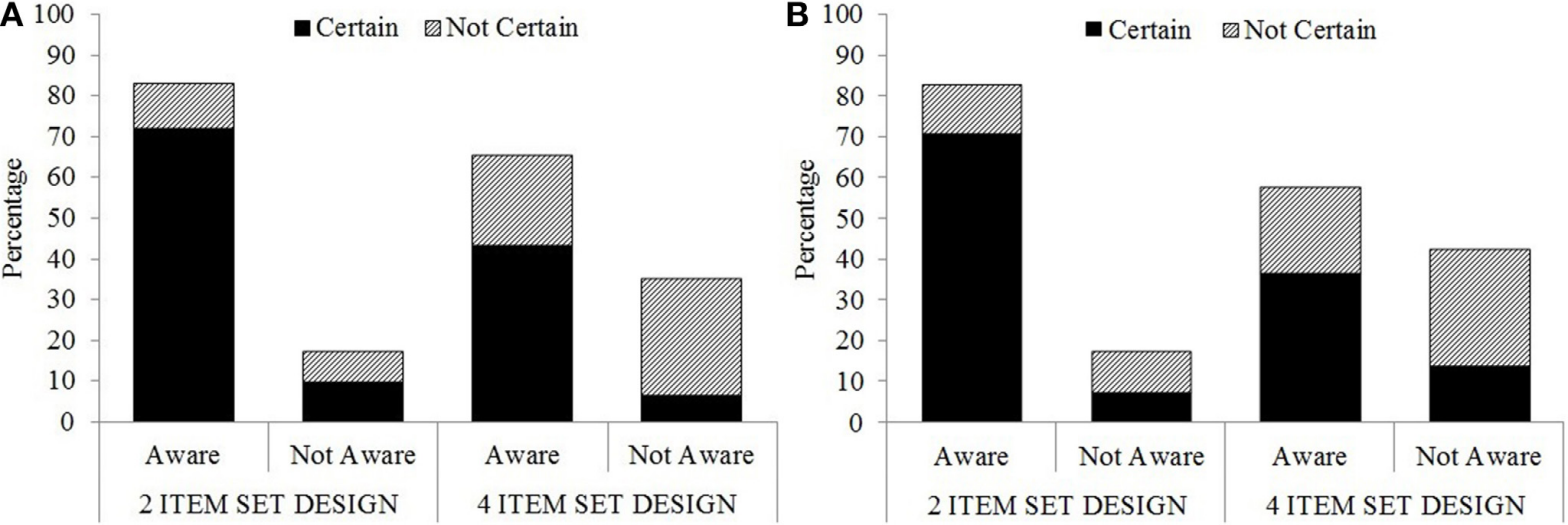
**Percentage of participants who were aware of the manipulation, and their confidence judgments in Experiment 1 **(A)** and Experiment 2 **(B)****.

Immediately after the experiments, participants answered a series of questions assessing their awareness of the ISPC manipulation (see Figure 7). Five participants' (3 participants in Experiment 1, 2 participants in Experiment 2) awareness data were not recorded. Regarding the first (awareness) question, in Experiment 1, 76 out of 103 (74%) participants reported noticing specific words and colors being paired more frequently. In other words, they noticed the ISPC manipulation. The number of participants who noticed the ISPC manipulation was higher for the 2-item set condition (44 out of 53, 83%) compared to the 4-item set condition (32 out of 50, 64%), χ^2^_(1)_ = 4.81, *p* < 0.05. Regarding the confidence judgments, 67 out of 102 (66%) participants reported that they were certain about their answers. This was higher for the 2-item set condition (43 out of 53, 81%) compared to the 4-item set condition (24 out of 49, 49%), χ^2^_(1)_ = 11.68, *p* < 0.001. The results for Experiment 2 regarding the awareness question were parallel to those of Experiment 1. Seventy-nine out of 111 (71%) participants reported noticing the ISPC manipulation. The number of participants who noticed the ISPC manipulation was higher for the 2-item set condition (48 out of 58, 83%) compared to the 4-item set condition (31 out of 53, 58%), χ^2^_(1)_ = 7.95, *p* < 0.005. Regarding the confidence judgments, 72 out of 111 (65%) participants reported that they were certain about their answer. This was higher for the 2-item set condition (46 out of 59, 78%) compared to the 4-item set condition (26 out of 52, 50%), χ^2^_(1)_ = 9.49, *p* < 0.005. To sum up, the number of participants who were aware of the ISPC manipulation, and who were certain about their responses, was higher for the 2-item-set condition compared to the 4-item-set condition (Figure 7)^3^.

**Figure 8:**
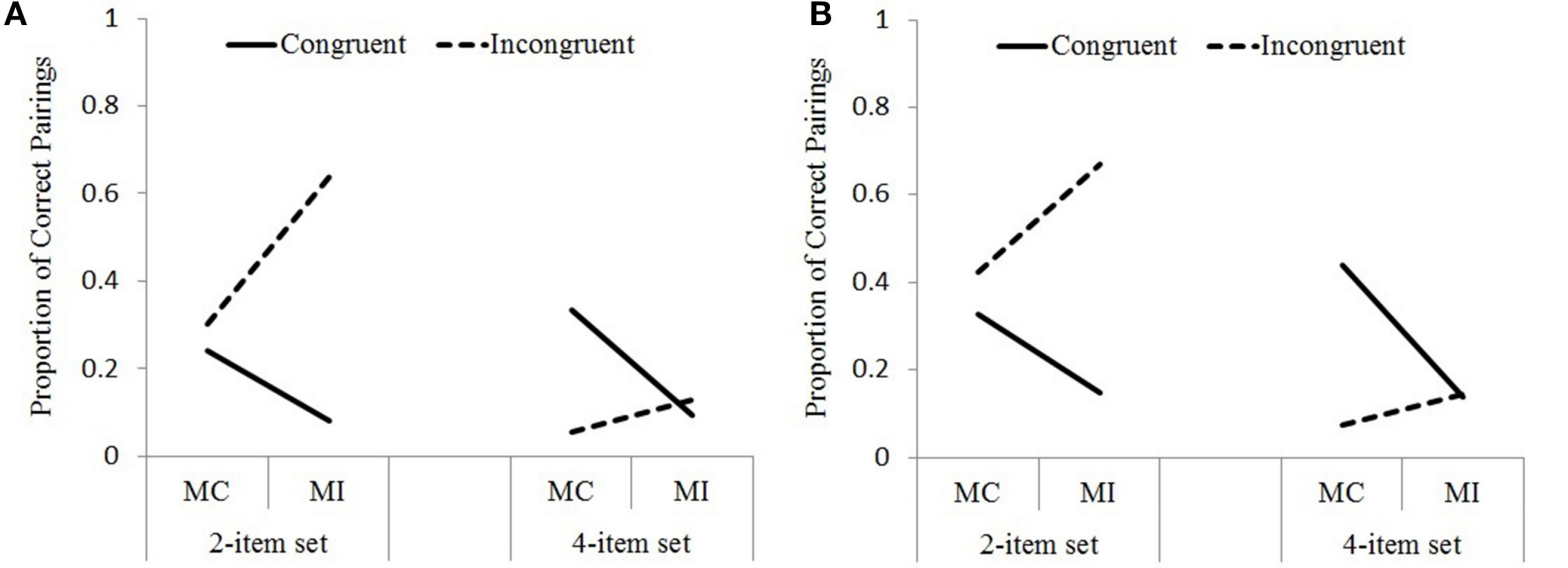
**Proportion of correct pairings for the matching question in Experiment 1 **(A)** and Experiment 2 **(B)****.

For the next (matching) question, participants were given 10 colors and 10 color words presented as two columns and were asked to connect the more frequently paired colors and color words by drawing a line in-between. Eight of the 10 colors and color words were used in the experiment, the remaining two were new. They were encouraged to guess if they were not certain, nevertheless, none of the participants paired the new colors and color words. For each participant, the *proportion of correct pairs* was calculated separately for the MC-congruent, MI-congruent, MC-incongruent, and MI-incongruent conditions (see Figure 8). Proportions were analyzed with separate 2X2 mixed-design ANOVAs for congruent and incongruent trials, with set size (2-item vs. 4-item) as the between-subjects factor, and proportion congruency (MC vs. MI) as the within-subject factor.

In Experiment 1, for the congruent pairs, the main effect of proportion congruency was significant, *F*_(1, 101)_ = 49.70, *MSE* = 0.041, *p* < 0.001, η^2^_p_ = 0.33. The proportion of correct MC-congruent pairs (0.29) was higher than the MI-congruent pairs (0.09). None of the other main effects or interactions were significant, *F*s < 2. For the incongruent pairs, there was a significant main effect of proportion congruency, *F*_(1, 101)_ = 49.89, *MSE* = 0.043, *p* < 0.001, η^2^_p_ = 0.33. The proportion of correct MI-incongruent pairs (0.38) was higher than the MC-incongruent pairs (0.18). There was a significant between-subjects main effect of set size, *F*_(1, 101)_ = 74.96, *MSE* = 0.098, *p* < 0.001, η^2^_p_ = 0.43. The proportion of correct incongruent pairs was higher for the 2-item set condition (0.47) compared to the 4-item set condition (0.09). The two-way interaction between set size and proportion congruency was also significant, *F*_(1, 101)_ = 20.06, *MSE* = 0.043, *p* < 0.001, η^2^_p_ = 0.17. The difference between the proportion of correct MI-incongruent and MC-incongruent pairs was more pronounced in the 2-item set condition compared to the 4-item set condition (2-item set MC = 0.30, 2-item set MI = 0.64, 4-item set MC = 0.06, 4-item set MI = 0.13).

Experiment 2 yielded results parallel to Experiment 1. For the congruent pairings, the main effect of proportion congruency was significant, *F*_(1, 110)_ = 60.57, *MSE* = 0.053, *p* < 0.001, η^2^_p_ = 0.36. The proportion of correct MC-congruent pairs (0.38) was higher than the MI-congruent pairs (0.14). The main effect of set size was not significant, *F* < 1. The two-way interaction between set size and proportion congruency was significant, *F*_(1, 110)_ = 4.04, *MSE* = 0.053, *p* < 0.05, η^2^_p_ = 0.04. The difference between the proportion of correct MC-congruent and MI-congruent pairs was more pronounced in the 4-item set condition compared to the 2-item set condition (2-item set MC = 0.33, 2-item set MI = 0.15, 4-item set MC = 0.44, 4-item set MI = 0.14). For the incongruent pairs, there was a significant main effect of proportion congruency, *F*_(1, 110)_ = 29.41, *MSE* = 0.048, *p* < 0.001, η^2^_p_ = 0.21. The proportion of correct MI-incongruent pairs (0.41) was higher than the MC-incongruent pairs (0.25). The between-subjects main effect of set size was significant, *F*_(1, 110)_ = 98.16, *MSE* = 0.109, *p* < 0.001, η^2^_p_ = 0.47. The proportion of correct incongruent pairs was higher for the 2-item set condition (0.55) compared to the 4-item set condition (0.11). The two-way interaction between set size and proportion congruency was also significant, *F*_(1, 110)_ = 9, *MSE* = 0.048, *p* < 0.005, η^2^_p_ = 0.08. The difference between the proportion of correct MI-incongruent and MC-incongruent pairs was more pronounced in the 2-item set condition compared to the 4-item set condition (2-item set MC = 0.42, 2-item set MI = 0.67, 4-item set MC = 0.07, 4-item set MI = 0.14).

In summary, the results for the matching question revealed that participants became aware of the congruent pairs more in the MC condition, and incongruent pairs more in the MI condition. Additionally, these differences between the MI-incongruent and MC-incongruent conditions were more pronounced in the 2-item set condition than the 4-item set condition (Figure 8).

### Delta plots

We compared the time course of ISPC effects for the 2-item and 4-item set conditions in Experiment 2 using delta plots (De Jong et al., 1994). Delta plots provide information on the ISPC effect across the RT distribution. In other words, they demonstrate how the ISPC effect changes as the responses slow down. Our previous average response latency analyses showed that the relationship between the ISPC effect and SOA was similar for the 2-item and 4-item set conditions. Parallel to this, we expected the time-course of the ISPC effect to be similar for the 2-item and 4-item conditions, as well.

**Figure 9:**
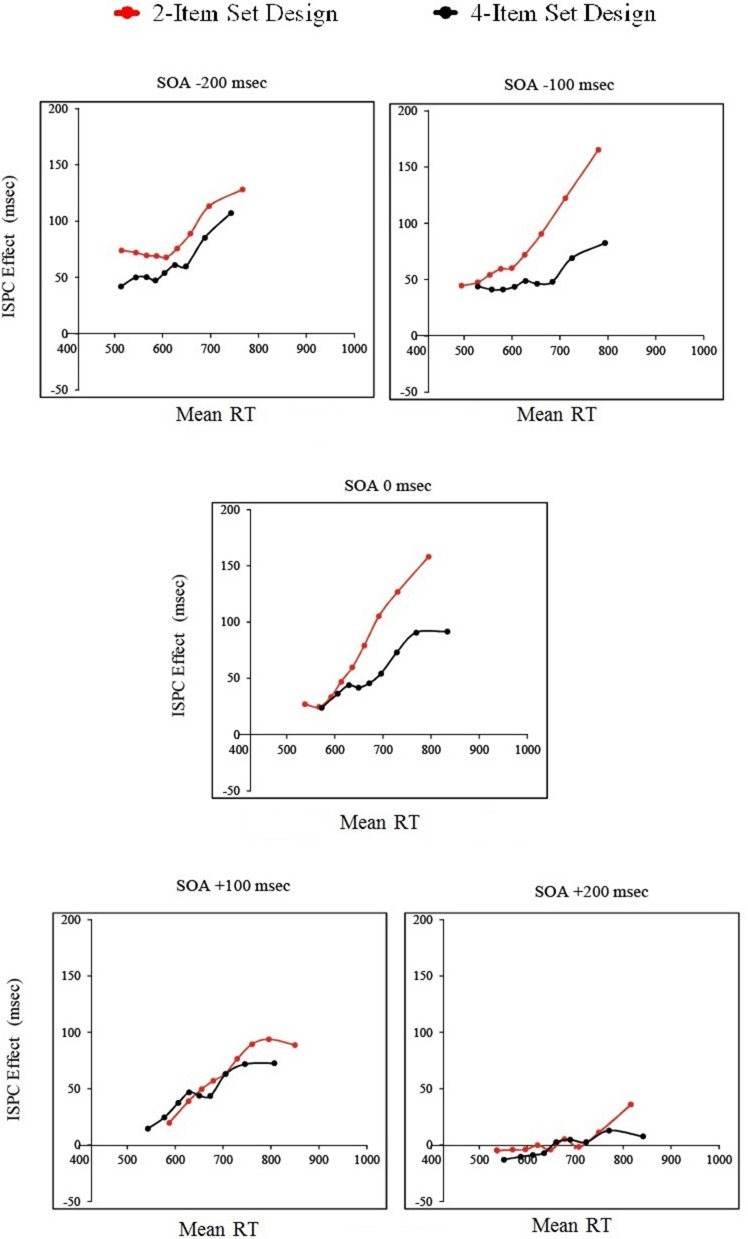
**Delta plots of the ISPC effects as a function of set size and SOA for Experiment 2**.

First the 10th, 20th… 80th, 90th percentiles of the correct RT data for each condition were calculated for each participant, and then averaged across participants. Then, ISPC effects were calculated for each SOA and each set size condition using these averaged percentiles, and are displayed on the vertical axis of the delta plots. Lastly, means of averaged percentiles were calculated, which are displayed on the horizontal axis of the plots (Figure 9).

Overall, the ISPC effect was smallest for the fastest responses and increased as the responses got slower. Delta plots for the 2-item and 4-item set conditions were more similar for the positive SOA conditions compared to the 0 SOA or negative SOA conditions. In the +200 SOA condition, the ISPC effect was absent in both the 2-item and 4-item set conditions, except for the slowest responses. In the +100 SOA condition, the time-course of the ISPC effect was similar for the 2-item and 4-item set conditions. In the 0 SOA and −100 SOA conditions, the ISPC effect for the 2-item and 4-item set conditions were similar for the fastest responses. However, for the slower responses, the ISPC effect in the 2-item set condition increased faster, compared to the 4-item set condition. In the -200 SOA condition, this pattern was reversed: for the fastest responses the ISPC effect for the 2-item set condition was larger compared to the 4-item set condition. However, they became more similar as the responses got slower.

When the delta plots for the different SOA conditions are compared within each set size condition, one can observe that while the time course of the ISPC effect across different SOA conditions are comparable for the 4-item set condition, they show more variation for the 2-item set condition. Indeed, the observed differences between the 2-item and 4-item set conditions across SOA conditions were driven primarily by the change in the delta plots for the 2-item set condition.

In summary, the delta plot analysis revealed that the ISPC effect was smaller for fastest responses and increased as the responses got slower. The ISPC effect followed different time courses for the 2-item and 4-item set conditions, with more stable time courses for the 4-item set condition compared to the 2-item set condition.

## Discussion

In the current study, we investigated the time course of the ISPC effect with two experiments by combining SOA and set size (Bugg and Hutchison, 2013) manipulations. More specifically, we manipulated the SOA between the relevant (color) and irrelevant (word) Stroop dimensions, and compared the change in the ISPC effect as a function of SOA for 2-item and 4-item set size conditions. In the first experiment, set size was manipulated as a between-subjects factor and SOA as a within-subject factor. In the second experiment, considering the possibility that participants might switch between control and contingency learning processes across different SOA blocks, SOA was manipulated as between-subjects factors. This also increased the number of stimuli for each SOA condition, improving the validity of observations. In addition to the conventional RT analyses, time course of the ISPC effect was investigated using delta plots. Furthermore, awareness data regarding the ISPC manipulations were collected and analyzed.

Overall, Experiment 1 and Experiment 2 did not yield different findings. The results indicated that the SOA manipulation was working as expected. In other words, the Stroop effect changed as a function of different SOAs (Glaser and Glaser, 1982). In addition to this, the SOA manipulation interacted with the ISPC effect. More specifically, the ISPC effects observed for different SOA conditions were comparable, except for the +200 SOA condition, in which the ISPC effect was very small, if present. Of specific interest to our study, the effect of the SOA manipulation on the ISPC effect did not change as a function of set size, as indicated by the lack of interaction between the ISPC effect, set size, and SOA.

Regarding the 2-item set condition, the results supported our predictions. The ISPC effect was observed, when participants were able to use the word to predict the correct response, yet it disappeared when the word was presented too late to help prediction, i.e., the +200 condition. The effects of word reading on color naming processes, nevertheless, persisted even in the +200 SOA condition, as indicated by a significant Stroop effect. This observation supports the notion that Stroop interference and contingency learning processes are independent in the 2-item set condition (Schmidt and Besner, 2008). Alternatively, it could also be argued that the word is presented too late to cause enough interference to call for control processes. This would also result in a reduced Stroop and ISPC effects, as observed^4^.

Results for the 4-item set condition were parallel to that of the 2-item set condition. If we attribute the observed ISPC effect in the 4-item set condition to item-specific control processes, then our results favor an explanation in which the word acts as the ISPC signal. The lack of an ISPC effect in the +200 SOA condition supports this explanation. Participants in this condition apparently initiate a response before the word has a chance to trigger item-specific control processes. Still, this explanation is somewhat difficult to accommodate, especially in the negative SOA conditions, since it requires controlling word reading processes *after* the word is read. Consequently, the observed ISPC effect in the 4-item set condition cannot be easily explained by reactive control processes.

Alternatively, the RT difference between the MC-incongruent and MI-incongruent trials may be a result of the differences in the frequency of the incongruent items, while the difference between the MC-congruent and MI-congruent trials may stem from differences in S-R contingency learning. Replicating these results in future studies using transfer stimuli is essential. Evidently, processes underlying the ISPC effect in the 4-item set condition need to be investigated in more detail to gain a better understanding of their exact nature.

Most ISPC explanations are based on computational models of the Stroop effect that assume spreading activation through color naming and word reading pathways in associative memory. These models erroneously predict that a larger Stroop effect would be observed when the word precedes the color (Cohen et al., 1990). The changes in the Stroop effect as a function of SOA have alternatively been explained with strategies that are independent of the Stroop effect itself (Glaser and Glaser, 1982; Cohen et al., 1990). According to these explanations; in separated versions of the Stroop task, participants rely on their knowledge of the relative timing of the relevant (color) and irrelevant (word) Stroop dimensions to modulate visual attention and/or response selection processes (Appelbaum et al., 2009, 2012). The participants in our study may have implemented a similar temporal attentional control strategy in addition to the S-R learning and/or item-specific control processes that they have been already using. Consequently, to account for the observed ISPC effect and SOA interaction, one has to either assume SOA related attentional control strategies or to adopt a different model of the Stroop effect (see Roelofs, 2010b).

A recent study argued that temporal learning might explain proportion congruency effects in the Stroop task (Schmidt, 2013c, 2014). More specifically Schmidt (2013c, 2014) argued that an explanation for the proportion congruency effects might be that the participants are learning, and in turn predicting, when to respond. Even though effects of temporal learning on the ISPC effect were not conclusive, there was a trend in the data of Schmidt (2014). Separated versions of the Stroop task provide the participants with more informative cues regarding when to respond. Therefore, in our experiment, a temporal learning mechanism might have played a role in the observed change in the magnitude of the ISPC effect as a function of SOA.

Additional to the conventional RT analyses, we investigated the time course of the ISPC effect using delta plots (De Jong et al., 1994). Overall, delta plot analyses showed that the ISPC effect was smaller for faster responses and increased as the responses got slower. The slopes of the delta plots for the 2-item set condition were steeper than those of the 4-item set condition, especially in the negative and 0 SOAs. On the one hand, it is reasonable to assume that S-R learning (or temporal learning) processes affect shorter RTs more than longer RTs since faster responses are more likely to be modified by S-R learning or temporal prediction processes. Attentional control processes, on the other hand, should affect longer RTs more than shorter RTs, since attentional effects are more likely to be cumulative (Schmidt, 2014). However, for both faster (as observed in the −200 SOA condition) and slower (as observed in −100 and 0 SOA conditions) responses, our analyses showed that whenever there was a difference between the 2-item and 4-item set conditions, the ISPC effect was larger for the 2-item set condition compared to the 4-item set condition. Therefore, the results do not fit well with the notion that S-R learning is dominant in the 2-item set condition and control processes are dominant in the 4-item set condition. This dissociation between the negative and positive SOA conditions regarding the change in the magnitude of the ISPC effect as a function of response latency, calls for further investigation.

To the knowledge of the authors, this study is the first in which, additional data were collected after an ISPC experiment to assess whether or not participants were aware of the ISPC manipulation. According to the results participants in the 2-item set condition were aware of the ISPC manipulation more than the participants in the 4-item set condition. They came up with a higher proportion of correct pairs in the experiment, as well. These results suggest that awareness might be playing a role in the observed differences between the 2-item and 4-item set ISPC effects. Previously, Crump et al. (2008) investigated the effects of awareness on the CSPC effect by explicitly telling the participants about the CSPC manipulation. Their results showed that awareness did not influence the CSPC effect. In a recent study, however, Blais et al. (2012) observed that awareness had little role in the list-wide PC effect. In the current study, we did not observe an effect of awareness on the magnitude of the ISPC effect, indicated by the results of our *post-hoc* analysis (see Footnote 3). Nevertheless, possible effects of awareness of the ISPC manipulation on the magnitude of the ISPC effect need to be investigated in more detail in order to gain a better understanding of the underlying mechanisms.

In conclusion, our results showed that manipulating the SOA between the relevant and irrelevant dimensions changed the ISPC effect. The ISPC effect observed in the +200 condition was smaller, if at all present, than the ISPC effects in other SOA conditions. Moreover, this pattern was observed in both the 2-item and 4-item set conditions, that is, regardless of whether reactive control or contingency learning processes were dominant. Furthermore, a higher percentage of participants were aware of the ISPC manipulation in the 2-item set condition compared to the 4-item set condition. In addition, RT distribution analyses (delta plots) revealed that the ISPC effect was smaller for fastest responses and increased as the responses got slower. The SOA manipulation proves promising to further the understanding of the mechanisms underlying the ISPC effect.

### Conflict of interest statement

The authors declare that the research was conducted in the absence of any commercial or financial relationships that could be construed as a potential conflict of interest.

## Footnotes

^1^We would like to thank our reviewer for this suggestion.

^2^We would like to thank James R. Schmidt for suggesting this analysis.

^3^In order to investigate whether subjects who were aware of the manipulation differed from subjects who weren't, we compared these two groups regarding the observed ISPC effects with non-parametric tests. Analyses did not yield significant results (*p*s > 0.05).

^4^We would like to thank our reviewer for this suggestion.
